# *Codonopsis pilosula* Polysaccharide (CPP) Alleviates D-Gal-Induced Aging and Gut Microbiota Dysbiosis

**DOI:** 10.3390/ijms27093933

**Published:** 2026-04-28

**Authors:** Bin Zhang, Chongyang Zhang, Miao Yu, Yudie Zhang, Xiangming Wang, Rongchang Chen, Xiaobo Sun

**Affiliations:** 1 Institute of Medicinal Plant Development, Peking Union Medical College, Chinese Academy of Medical Sciences, Beijing 100193, China; zhangbin7@126.com (B.Z.); zcy122999@163.com (C.Z.);; 2 State Key Laboratory for Quality Ensurance and Sustainable Use of Daodi Herbs, Beijing 100193, China; 3 Diabetes Research Center, Chinese Academy of Medical Sciences, Beijing 100193, China; 4 Key Laboratory of Efficacy Evaluation of Chinese Medicine against Glyeolipid Metabolism Disorder Disease, State Administration of Traditional Chinese Medicine, Beijing 100193, China; 5 Department of Cell Biology, School of Basic Medical Sciences, Capital Medical University, Beijing 100069, China; xiangming@ccmu.edu.cn

**Keywords:** *Codonopsis pilosula* polysaccharide, inflammaging, inflammatory response, cognitive function, gut microbiota

## Abstract

As a traditional tonic in Chinese herbal medicine, *Codonopsis pilosula* exerts anti-aging effects, but studies on *Codonopsis pilosula* polysaccharides (CPPs) in the regulation of gut microbiota dysbiosis and the associated pathways remain limited. This study explored CPP’s anti-aging effects and mechanisms using a D-galactose-induced aging mouse model. In vivo results showed that CPP improved cognitive deficits, alleviated systemic aging, reduced neuroinflammation/oxidative stress, mitigated pathological tissue changes, and inhibited aging markers (p53, p21, and p16). Transcriptomic/metabolomic analyses indicated that CPP regulated inflammation-related genes and metabolites, with anti-inflammatory effects mediated via the MAPK pathway. 16S rRNA sequencing revealed that CPP restored gut microbiota diversity. In vitro experiments confirmed CPP’s anti-aging effects and identified the MAPK/FOXO1 pathway as a potential target. In conclusion, CPP exhibits potential anti-aging effects, possibly through the MAPK pathway and gut microbiota modulation.

## 1. Introduction

Aging is a complex and ongoing biological process influenced by various factors. At the cellular and molecular levels, key mechanisms include persistent inflammation, oxidative damage, mitochondrial dysfunction, genomic instability, shortening of telomeres, and disruptions in cellular signaling pathways [1]. In recent years, chronic inflammation has emerged as a major focus in aging research, being recognized as a hallmark of aging and a critical driver of the aging process [2,3]. Under normal physiological conditions, the inflammatory response serves as a protective mechanism against injury and infection, typically self-limiting and tightly regulated. However, with advancing age, shifts in the internal physiological milieu can lead to a state of chronic, low-grade inflammation [4].

Inflammation significantly accelerates aging through multiple mechanisms. Senescent cells secrete cytokines, chemokines, and proteases, known as the senescence-associated secretory phenotype (SASP), which sustains immune activation and results in persistent low-grade inflammation [5,6]. SASP factors, such as IL-6 and TNF-α, disrupt nearby cell function and metabolism while recruiting immune cells that amplify local inflammatory responses [7]. Furthermore, inflammation-induced oxidative stress increases reactive oxygen species (ROS) production, damaging biomacromolecules, impairing cell proliferation, inducing apoptosis, and ultimately promoting cellular senescence [8]. Chronic inflammation also activates glial cells, triggering neuroinflammation that disrupts neuronal communication and accelerates cognitive decline and neurodegenerative diseases such as Alzheimer’s disease (AD) and Parkinson’s disease [9,10]. Notably, the D-galactose-induced aging model, which is widely used in our study, can effectively recapitulate the above-mentioned aging-related pathological features, and recent studies have shown that CPP can protect against brain aging in this model by alleviating oxidative damage and neuroinflammation [11,12], providing further support for the rationale of our experimental model selection.

*Codonopsis pilosula*, a valuable herb in traditional Chinese medicine (TCM), has attracted increasing scientific interest for its polysaccharide components, which exhibit diverse biological activities due to their unique chemical structure. Modern pharmacological studies indicate that CPP possesses immunomodulatory, anti-inflammatory, neuroprotective, antioxidant, and anticancer properties. In neurodegenerative contexts, CPP can mitigate memory decline in AD models by inhibiting the BACE1 activity [13]. It also reduces Tau hyperphosphorylation via PP2A activation and alleviates cognitive impairment by restoring synaptic plasticity [14]. In addition, CPP can alleviate Aβ1-40-induced neuron damage through maintaining energy metabolism homeostasis, thereby delaying AD progression [15].

In aging and inflammatory models, CPP can ameliorate lung pathology in D-gal-induced aging mice [16] and protect against DSS-induced colitis by modulating gut microbiota, enhancing short-chain fatty acid production, and balancing anti- and proinflammatory cytokines related to the Th17/Treg homeostasis [17]. Notably, the regulatory role of CPP in intestinal immunity, which plays a key role in gut microbiota homeostasis, has been gaining increasingly attention, and recent studies have confirmed that CPP can regulate intestinal immune function and shape gut microbiota composition to exert systemic anti-inflammatory and anti-aging effects [18]. CPP appears to counteract chronic inflammation-induced aging through dual mechanisms: modulating immune balance to suppress proinflammatory SASP factors and reducing oxidative damage by scavenging excess ROS. These actions help stabilize cellular function and metabolism, thereby slowing cellular aging [19,20,21].

CPP exhibits potential anti-inflammatory and anti-aging properties; however, several specific gaps in current research remain to be filled, which is the core focus of the present study. Although previous studies have documented the anti-inflammatory and neuroprotective effects of CPP, as well as its regulatory role in the gut microbiota and intestinal immunity, the specific molecular mechanism linking CPP-mediated gut–brain axis modulation to neuroinflammation inhibition has not been fully elucidated. In particular, the rationale for focusing on the MAPK/FOXO1 signaling pathway in this regulatory process is not sufficiently articulated. Accumulating evidence has demonstrated that the MAPK/FOXO1 pathway serves as a key molecular bridge connecting peripheral intestinal immune responses and central neuroinflammation, and its dysregulation is closely associated with age-related cognitive decline and neuroinflammation progression [22,23]. This pathway not only participates in the regulation of intestinal immune cell activation and proinflammatory cytokine secretion but also mediates the transmission of gut-derived inflammatory signals to the central nervous system, thereby affecting neuronal function and accelerating brain aging. Given that CPP can modulate the gut microbiota and intestinal immunity to exert systemic anti-inflammatory effects, it is reasonable to hypothesize that CPP may regulate neuroinflammation and ameliorate aging through the MAPK/FOXO1 signaling pathway, a mechanism that has not been previously explored. This D-gal-induced aging model effectively simulates chronic inflammation and the physiological–pathological changes associated with natural aging, providing a robust platform for investigating the anti-aging effects of CPP and clarifying the above-mentioned mechanism. In the current study, we systematically evaluated the protective roles of CPP in D-gal-induced aging mice through observation of learning and memory ability, inflammatory factors, and pathological changes in the brain, muscle, and liver. Through integrated transcriptomic and metabolomic analyses, we confirmed that CPP ameliorates aging primarily by suppressing neuroinflammation via the MAPK/FOXO1 signaling pathway. These findings provide a novel target for innovative drug development against aging and offer new strategies for its clinical treatment.

## 2. Results

### 2.1. CPP Improves Cognitive and Memory Dysfunction in Aging Mice Induced by D-Gal

**Figure 1:**
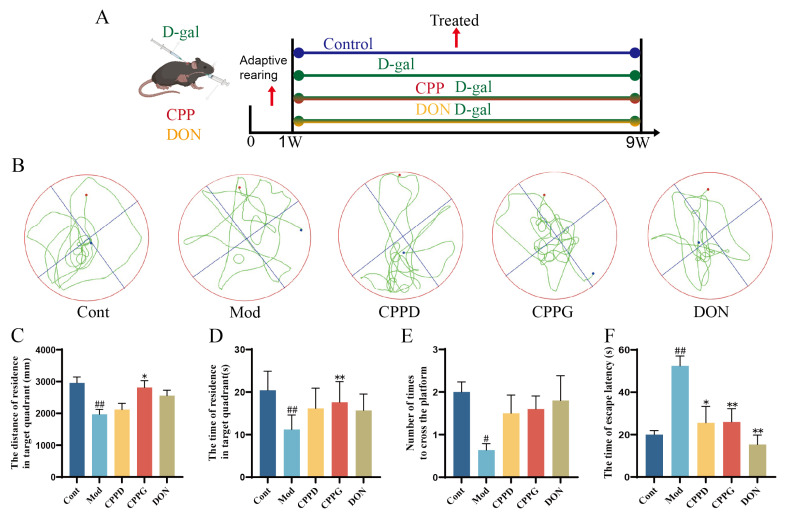
CPP improves cognitive abilities as demonstrated by the Morris water maze test. (**A**) A diagram showing the experimental arrangement. (**B**–**F**) Illustrations of movement trajectories, total distance covered, time spent, frequency of platform crossings, and latency observed during the water maze evaluation. The data are expressed as the mean ± SEM *(n (n* = 5). # *p* < 0.05, ## *p* < 0.01 compared with the control group; * *p* < 0.05, ** *p* < 0.01 compared with the model group.

Figure 1A provides a detailed schematic that illustrates the chronological development of our methodological approach. To evaluate the alterations in spatial learning and memory in aging mice, as well as the therapeutic impact of CPP, we conducted the Morris water maze test, as shown in Figure 1B. The movement patterns of mice in the Mod group were more erratic compared to those in the Cont group, indicating their difficulty in independently locating the platform. During the behavioral evaluation, some mice exhibited atypical movement behaviors, characterized by uncoordinated swimming. Additionally, these mice visited the target quadrant with the submerged escape platform significantly less often than the control group. The mice were allowed to swim freely in the water basin while searching for the platform, consistently showing a preference for the quadrant containing the platform. Mice in the DON group concentrated on the platform quadrant upon entering the water but also explored other areas. In comparison to the Mod group, the CPP group mice took longer to reach the target quadrant (Figure 1C), spent more time there (Figure 1D), crossed the platform fewer times (Figure 1E), and exhibited a notably longer escape latency (Figure 1F).

### 2.2. CPP Alleviates Systemic Aging and Reduces Neuroinflammation and Oxidative Stress Levels

**Figure 2:**
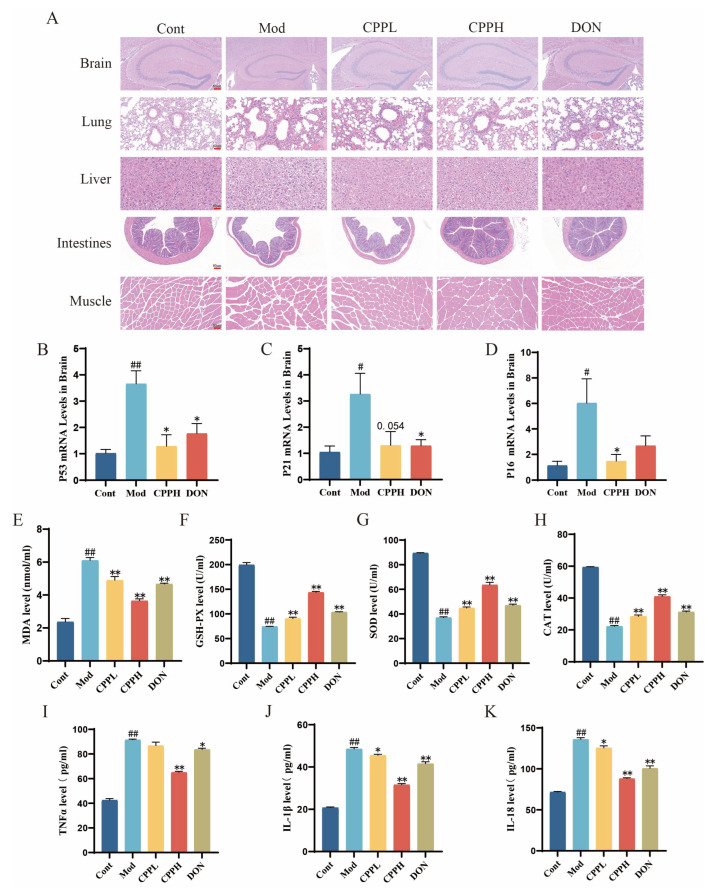
The effects of CPP on systemic organs and inflammation in aging mice. (**A**) Sample images showing HE staining of brain, lung, liver, muscle, and intestinal tissues. (**B**–**D**) PCR analysis of aging marker genes *P53, P21*, and *P16* in brain tissues *(n* = 3). (**E**–**H**) Evaluation of serum levels of oxidative stress markers MDA, GSH-PX, SOD, and CAT. (**I**–**K**) Measurement of serum levels of inflammatory factors TNF-α, IL-1β, and IL-18. The results are shown as the mean ± SEM *(n* = 5). # *p* < 0.05, ## *p* < 0.01 compared with the control group; * *p* < 0.05, ** *p* < 0.01 compared with the model group.

As shown in Figure 2A, the control group consisted of hippocampal neurons that were densely packed, uniformly shaped, and evenly spaced apart. In contrast, the model group exhibited misaligned neurons with irregular shapes, shrunken nuclei, some loss of nuclei, and larger gaps between cells. Treatment with CPP significantly improved these pathological conditions compared to the model group, as evidenced by better tissue organization, more regular cell shapes, normalized nuclei, and reduced intercellular spacing, indicating less neuronal damage. In the lung tissues, the control group had intact alveolar structures and normal bronchial morphology, while the model group showed thickened alveolar walls, disrupted architecture, and significantly widened septa. The liver tissue in the control group appeared normal with orderly arranged cells, whereas the model group exhibited clear ballooning degeneration, swollen hepatocytes, and infiltration of inflammatory cells. The muscle tissue in the control group was also normal, with consistent shapes and sizes, while the model group had deformed and swollen muscle cells, interstitial fibrosis, and enlarged intercellular spaces. Compared to the model treatment, high-dose CPP (CPP-H) treatment significantly improved the pathological changes in the lung, liver, and muscle tissues. Furthermore, PCR analysis indicated that the aging markers P53, P21, and P16 were elevated in the model group, but the administration of CPP-L and CPP-H significantly reduced these markers (Figure 2B–D), with CPP-H proving to be more effective than CPP-L. As a result, further research was focused on CPP-H, which is now referred to as CPP.

As illustrated in Figure 2E–K, the model group exhibited significantly lower activities of serum antioxidant enzymes (CAT, SOD, and GSH-Px) compared to the control group, along with a marked increase in MDA levels, which suggests considerable oxidative damage and diminished antioxidant capacity. Furthermore, the serum concentrations of proinflammatory cytokines (TNF-α, IL-1β, and IL-18) were significantly higher in the model mice than in the control mice. In contrast, both the CPP- and DON-treated groups showed enhanced antioxidant defenses, as evidenced by elevated levels of oxidative stress markers and a significant decrease in inflammatory mediator levels. Overall, these results indicate that CPP effectively boosts antioxidant mechanisms and mitigates excessive inflammatory responses in aging mouse models.

### 2.3. Transcriptomic Analysis Uncovers the Important Biological Processes and Signaling Pathways Through Which CPP Safeguards the Brain’s Learning and Cognitive Abilities

**Figure 3:**
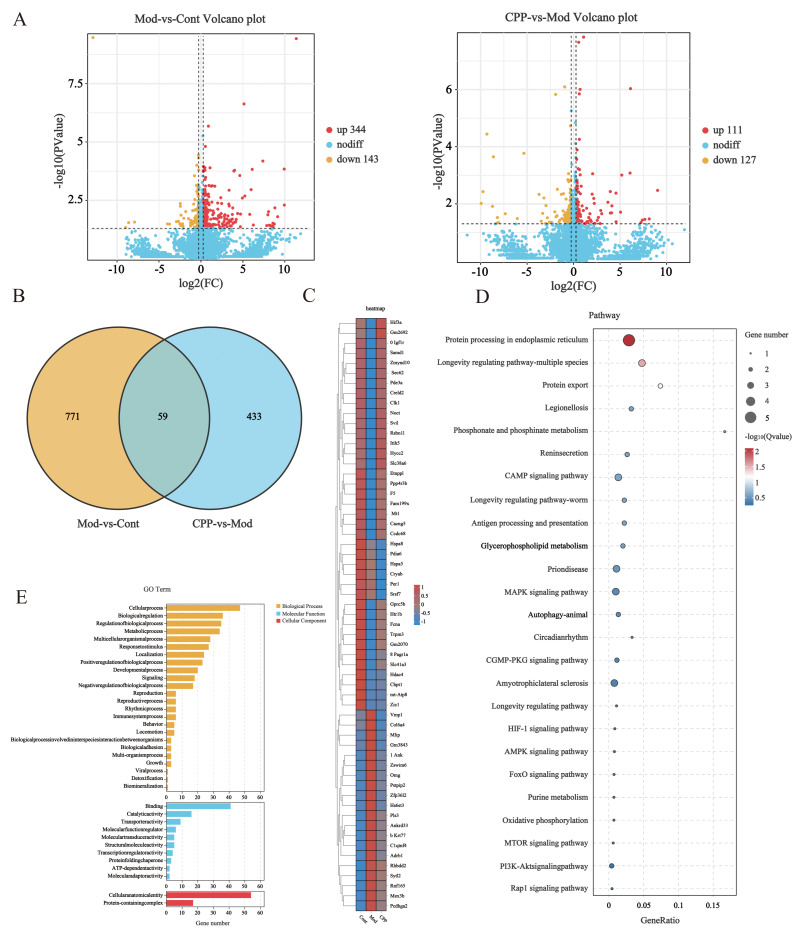
Transcriptomic analysis of brain tissues from D-gal-induced aging mice treated with CPP. (**A**) Volcano plot of differential genes in Mod-vs-Cont and CPP-vs-Mod (differential expression analysis was performed using DESeq2, and differential genes were screened under the conditions of |log2fold change| > log2(1.5) and *p*-value < 0.05). (**B**) Venn diagram of differentially expressed genes in Mod-vs-Cont and CPP-vs-Mod. (**C**) Heatmap of differentially expressed genes. (**D**) Bubble plot of KEGG signaling pathways enriched with differentially expressed genes. (**E**) GO term enrichment analysis workflow highlighting significant biological processes associated with the DEGs.

Differential expression analysis was performed using DESeq2, and differential genes were screened under the criteria of |log2foldchange| > log2(1.5) and *p*-value < 0.05 (Figure 3A). In the Mod-vs-Cont comparison group, 830 differential genes were identified, including 83 upregulated genes and 747 downregulated genes. In the CPP-vs-Mod comparison group, 492 differential genes were screened out, with 32 upregulated genes and 461 downregulated genes. The 492 differential genes between the CPP group and the Mod group were intersected with disease-related genes from the Mod-vs-Cont comparison group, resulting in a set of disease-related genes ameliorated by CPP, which contained a total of 59 differential genes, referred to herein as the CPP set (Figure 3B,C). Among them, 20 genes were upregulated in Mod-vs-Cont and downregulated in CPP-vs-Mod, while 34 genes were downregulated in Mod-vs-Cont and upregulated in CPP-vs-Mod. To understand the relevant cellular processes and functions of these differential genes, GO enrichment analysis was conducted on the 59 differential genes (Figure 3E). The results showed that these differential genes were mainly enriched in biological processes such as ion binding, transporter activity, and cation binding. This suggests that the proteins encoded by the differential genes may be involved in the occurrence and development of diseases through pathways such as ion homeostasis regulation, the action of aging-related transporters, abnormal cation metabolism, abnormal signal transduction, or dysfunction of related proteins. To further confirm the biological pathways mediated by these genes, KEGG pathway enrichment analysis was performed on the genes in this set (Figure 3D). Among them, “protein processing in endoplasmic reticulum” and “longevity regulating pathway” were the most significantly enriched. The set of differential genes may mainly affect protein homeostasis and core regulatory pathways of aging. Among them, studies have shown that genes including Pstpip2 [24], C1qtnf4 [25], OMG [26], Zfp36l2 [27], Ankyrin [28], Pls3 [29], Sytl2 [30], Mex3b [30] and Vmp1 [31] are involved in inflammatory responses through mechanisms such as immune regulation, signal pathways, and tissue damage repair.

### 2.4. Analysis of Serum Metabolomics Uncovers the Significant Biological Processes and Signaling Pathways Through Which CPP Slows Down the Aging Process

**Figure 4:**
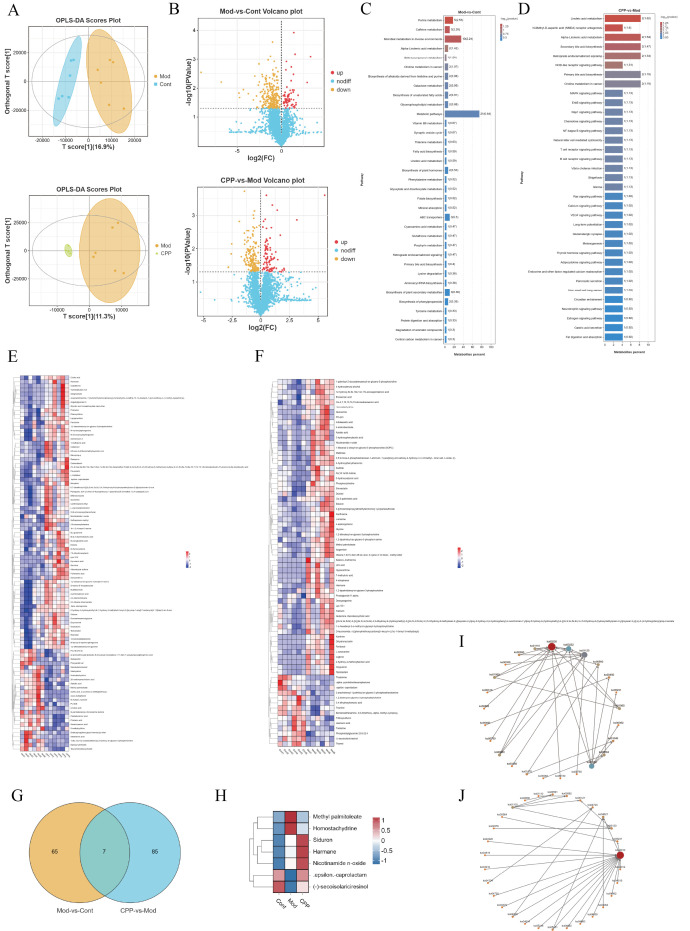
Results of the serum metabolomics analysis of D--gal--induced aging mice treated with CPP. (**A**) OPLS-DA score plots comparing Mod-vs-Cont and CPP-vs-Mod. (**B**) Volcano plots showing differentially abundant metabolites in Mod-vs-Cont and CPP-vs-Mod. (**C**) Bar chart illustrating KEGG signaling pathways enriched with differentially abundant metabolites in Mod-vs-Cont. (**D**) Bar chart depicting KEGG signaling pathways enriched with differentially abundant metabolites in CPP-vs-Mod. (**E**) Heatmap representing differentially abundant metabolites in Mod-vs-Cont. (**F**) Heatmap of differentially abundant metabolites in CPP-vs-Mod. (**G**) Venn diagram of differentially abundant metabolites in both Mod-vs-Cont and CPP-vs-Mod. (**H**) Heatmap of seven differentially abundant metabolites common to Mod-vs-Cont and CPP-vs-Mod. (**I**) Interaction network diagram of KEGG-enriched pathways for the Mod-vs-Cont comparison group. (**J**) Interaction network diagram of KEGG-enriched pathways in the CPP-vs-Mod comparison group.

To examine the changes in the serum metabolome during CPP treatment and its potential to slow aging, we conducted a nontargeted metabolomics analysis on serum samples from each group using LC‒MS technology. By identifying and quantifying metabolites and evaluating the quality of the raw data, we detected a total of 4582 metabolites, with 2646 identified in positive ion mode and 1936 in negative ion mode. We applied orthogonal partial least squares discriminant analysis (OPLS-DA) to compare the metabolites across both ion modes, resulting in OPLS-DA score plots (Figure 4A). The results showed a distinct separation between the Cont group and the Mod group, as well as between the Mod group and the CPP group, indicating significant differences in metabolites among the groups. In the Mod group, we identified 72 differentially abundant metabolites compared to the Cont group, with 14 significantly upregulated and 58 significantly downregulated. In the CPP group, we found 92 differentially abundant metabolites compared to the Mod group, with 25 significantly upregulated and 67 significantly downregulated (Figure 4B,E,F). Additionally, 92 differentially abundant metabolites were identified between the CPP and Mod groups. The overlap of these metabolites with those associated with diseases revealed a set of seven disease-related metabolites affected by CPP (Figure 4G,H). To further investigate the biochemical metabolic pathways and signaling pathways linked to these differentially abundant metabolites, we performed a metabolite pathway enrichment analysis for the Mod-vs-Cont and CPP-vs-Mod comparison groups using KEGG (Figure 4C,D). We selected the top 35 significantly enriched pathways based on their *p* values and created a network diagram to illustrate the interactions between different pathways, helping to identify core pathways (Figure 4I,J). Among the KEGG signaling pathways enriched in the CPP-vs-Mod comparison group, the MAPK signaling pathway (ko04010) was found to interact with other pathways and contained the highest number of enriched metabolites.

### 2.5. A Joint Analysis of Transcriptomes and Metabolomes Uncovers the Signaling Pathways That Contribute to the Anti-Inflammatory Effects of CPP in Older Mice

The inflammation pathways in the brain are closely linked to the circulatory system. When inflammation takes place in the brain, it triggers NF-κB, resulting in the activation of proinflammatory genes. The products of these genes may either pass through the blood–brain barrier or affect the peripheral immune system, changing the concentrations of inflammation-related substances (like cytokines and acute-phase proteins) in the blood. Conversely, inflammatory signaling molecules present in the blood can also influence gene expression in the brain and adjust the neuroimmune response.

**Figure 5:**
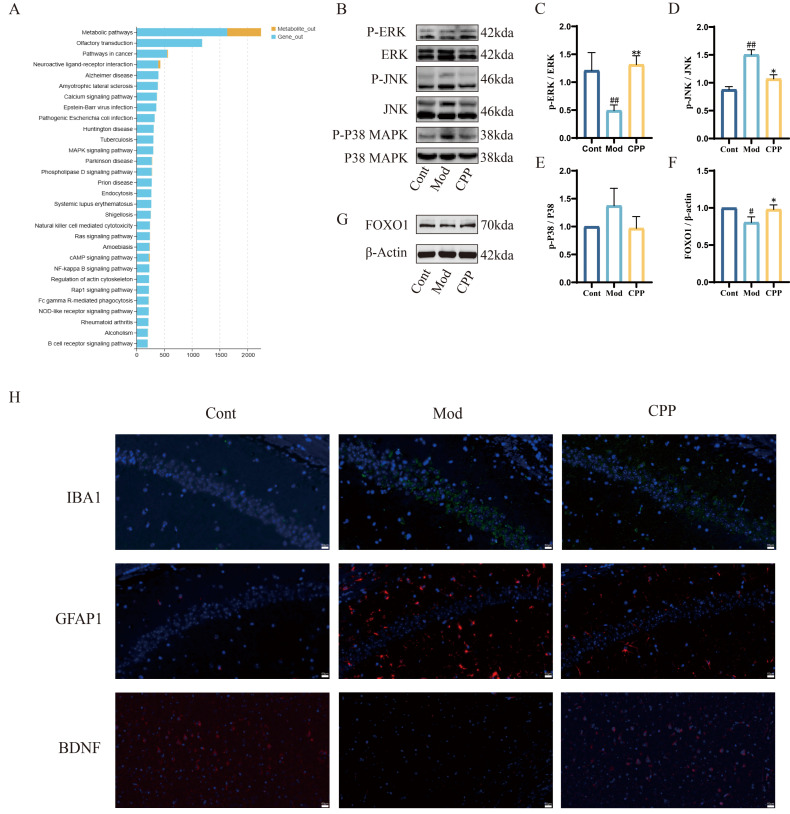
Combined transcriptome and metabolome analysis of the anti-inflammatory effect of CPP in aging mice. (**A**) Bar chart of the KEGG signaling pathways associated with the combined transcriptome and metabolome analysis. (**B**) The expression and phosphorylation of proteins related to the MAPK signaling pathway in brain tissues were detected by Western blotting (WB). The protein bands corresponding to P-ERK (42 kDa), ERK (42 kDa), P-JNK (46 kDa) and JNK (46 kDa), as well as P-P38 MAPK (38 kDa) and P38 MAPK (38 kDa), in the Cont, Mod, and CPP-H groups are shown from top to bottom. (**C**–**E**) Statistical analysis of the ratio of the phosphorylation level to the total protein level of proteins related to the MAPK signaling pathway. (**G**) The protein expression of FOXO1 (70 kDa) in brain tissues was detected by WB. (**F**) Statistical analysis of the ratio of the protein levels of FOXO1 to the total protein levels. (**H**) Immunohistochemical staining showing the distribution of IBA1, GFAP, and BDNF in the brain tissues of the Cont, Mod, and CPP-H groups. # *p* < 0.05, ## *p* < 0.01 compared with the control group; * *p* < 0.05, ** *p* < 0.01 compared with the model group.

To investigate this, we conducted KEGG enrichment analysis on the serum metabolome and brain transcriptome of the CPP group, correlated the findings, and compared the KEGG pathways that were enriched in both groups. We identified 208 common pathways (Table S2), with the MAPK signaling pathway being notably enriched (Figure 5A). Consequently, we identified proteins linked to the MAPK signaling pathway in brain tissues.

The JNK and p38 MAPK pathways, when triggered by oxidative stress and inflammatory signals, not only become activated but also enhance the production of inflammatory factors, leading to a detrimental cycle that accelerates brain tissue aging and neurodegeneration. As the aging process progresses, improper regulation of the ERK pathway may impact neural plasticity, which in turn affects learning and memory. Western blotting (WB) results showed that the model group of mice exhibited increased phosphorylation levels of JNK and p38 MAPK, while ERK phosphorylation levels were decreased. Additionally, the expression of several proteins was significantly modified by CPP, aligning more closely with those in the control group (Figure 5B–E).

Numerous studies have established a strong link between FOXO and aging [32]. Additionally, FOXO, a downstream effector of the MAPK pathway, was studied in brain tissues. The findings showed a marked reduction in FOXO protein levels in older mice, which was significantly restored by CPP treatment (Figure 5G,F). Additionally, we observed that CPP significantly lowered the levels of IBA1 and GFAP in brain tissues triggered by D-gal and enhanced BDNF expression (Figure 5H).

These results indicate that CPP may mitigate the cognitive decline associated with aging by inhibiting inflammation mediated by the MAPK/FOXO pathway.

### 2.6. CPP Ameliorates Gut Microbiota Dysbiosis in D-Gal-Induced Aging Mice

**Figure 6:**
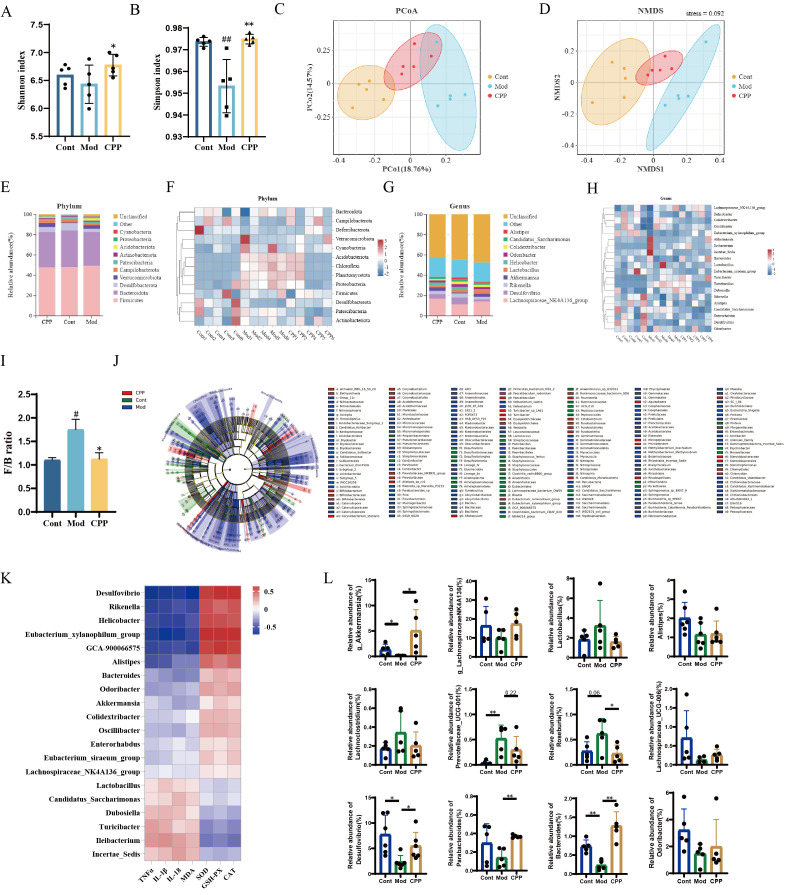
CPP alters the gut microbiota composition in D-gal-induced aging mice. (**A**,**B**) α-diversity analysis, with microbiota diversity derived from Shannon and Simpson’s evenness indices. (**C**,**D**) PCoA and NMDS plots illustrating the gut microbiota community within the OUT group. (**E**) Phylum-level abundance of the gut microbiota in the intestines. (**F**) Phylum-level heatmap of the gut microbiota in feces. (**G**) Genus-level abundance of the gut microbiota in feces. (**H**) Genus-level heatmap of the gut microbiota in feces. (**I**) F/B ratio *(n* = 6). (**J**) Taxonomic cladogram of significantly different taxa analyzed by LEfSe. (**K**) Pearson correlation analysis of the gut microbiota significantly altered at the genus level with inflammatory factors and antioxidant indicators. (**L**) Quantitative analysis of the microbiota associated with short-chain fatty acids. The data are expressed as the means ± SEM *(n* = 5). # *p* < 0.05, ## *p* < 0.01 vs. the Cont group; * *p* < 0.05, ** *p* < 0.01 vs. the Mod group.

The gut microbiota composition in fecal samples was examined using 16S rRNA sequencing. The Mod group showed a notable decrease in bacterial diversity, as indicated by the Shannon and Simpson indices, compared to the Cont group, but this reduction was significantly reversed with CPP treatment (Figure 6A,B). PCoA and NMDS analyses indicated clear differences in the gut microbiota profiles among the Cont, Mod, and CPP-treated mice (Figure 6C,D). Further examination at the phylum and genus levels (Figure 6E–H) showed an increased Firmicutes-to-Bacteroidetes (F/B) ratio in the Mod group relative to the Cont group, which was normalized following CPP treatment (Figure 6I). LEfSe analysis identified distinct microbial taxa across the three groups, illustrated in a taxonomic tree (Figure 6J). Specifically, 39, 155, and 37 taxa were found in the Cont, Mod, and CPP-treated groups, respectively, with significant taxonomic differences. Additionally, we conducted Pearson correlation analysis on the top twenty microbiota in the ELISA and created a gut microbiota genus heatmap (Figure 6K). The analysis indicated that most gut microbiota had positive correlations with proinflammatory cytokines (TNF-α, IL-1β, and IL-18) and negative correlations with antioxidant markers (SOD, GSH-PX, and CAT). These results suggest that the gut microbiota may influence the inflammatory response and oxidative stress associated with CPP. Our study also detected significant changes in tight junction proteins and TNF-α levels in intestinal tissue, which were notably improved by CPP (Figure S1). Quantitative analysis showed that certain short-chain fatty acid-producing bacteria [33], including *Akkermansia, Alistipes, Odoribacter, Bacteroides, Desulfovibrio, Lachnospiraceae_UCG-006*, and *Lachnospiraceae_NK4A136*_group, were less abundant in the Mod group (Figure 6L). Furthermore, significant alterations in acetic acid levels were observed in the serum (Figure S2). These results imply that D-galactose may induce microbial dysbiosis and contribute to aging in mice. Overall, the findings indicate that CPP effectively ameliorates gut microbiota dysbiosis in aged mice.

## 3. Discussion

In this study, we systematically explored several critical aspects. First, CPP treatment was effective in mitigating cognitive impairment and reducing inflammatory biomarkers in D-gal-induced senile mice, as evidenced by the Morris water maze results. The model group exhibited altered learning and memory capacity, whereas CPP-treated mice presented enhanced cognitive functions, with evident reductions in tissue damage at high doses. Second, CPP may alleviate inflammatory aging through modulation of the gut–brain axis. Mechanistic studies revealed that CPP regulates the composition and diversity of the gut microbiota, restores the intestinal bacterial ecosystem, and influences neurotransmitter synthesis and release via gut microbiota metabolites, thereby affecting cognitive function and emotional states. Third, CPP reduces the impact of inflammation on cognitive impairment in aging mice by modulating the MAPK and FOXO pathways. Specifically, CPP can modulate the expression of related genes, affect the phosphorylation levels of proteins in the MAPK pathway, and play a role in anti-inflammatory and anti-aging effects. Accordingly, CPP may be a promising and potential drug for treating aging-related diseases, especially those involving cognitive impairment and inflammation.

However, it should be emphasized that further research is needed to confirm its efficacy and safety in clinical settings and to fully elucidate the molecular regulatory network involved.

The aging process is generally accompanied by chronic inflammation, disorders of the immune system and organ dysfunction. The disruption of inflammatory markers and oxidative stress biomarkers in serum plays a central role in both aging and disease pathogenesis. Oxidative stress leads to oxidative damage to biological macromolecules, causing endogenous damage and cytokine release in the body, resulting in inflammaging [34], which in turn leads to cognitive impairment. Recent studies have identified reactive oxygen species (ROS) from intracellular or mitochondrial sources as being involved in hippocampal (neuronal) dysfunction at physiological concentrations [8]. As signaling molecules, ROS can activate various inflammatory signaling pathways, such as the MAPK signaling pathway [35], triggering the production of various proinflammatory cytokines [36]. Therefore, reducing the production of MDA while increasing the enzymatic activity of SOD, CAT, and GSH-Px can effectively decrease the activation of reactive oxygen species (ROS), mitigate oxidative stress and modulate the inflammatory response. In our study, we demonstrated that D-gal significantly induces systemic aging. To elucidate the molecular mechanism underlying this process, we comprehensively analyzed oxidative stress and inflammatory states in the serum through the detection of key biomarkers. Specifically, we measured oxidative stress indicators, including CAT, SOD, GSH-Px, and MDA, as well as proinflammatory cytokines, such as TNF-α, IL-1β, and IL-18. Our findings revealed that CPP had a pronounced effect on oxidative stress pathways, effectively reducing MDA levels while increasing CAT, SOD, and GSH-Px activity. Furthermore, the proinflammatory cytokines TNF-α, IL-1β, and IL-18, which characterize the senescence-associated secretory phenotype (SASP), were identified as key mediators of chronic inflammation and senescence promotion in normal cells. The administration of CPP significantly decreased the levels of TNF-α, IL-1β, and IL-18, thereby inhibiting the production of inflammatory factors in the body. These findings indicate that CPP may exert a protective effect on aging-related inflammation and oxidative stress through the modulation of these critical signaling pathways. These results suggest that CPP may delay aging and improve cognitive dysfunction by inhibiting oxidative stress and reducing inflammation.

Aging is a complex process involving changes in multiple systems and metabolism. As multi-omics characterization methods, transcriptomics and metabolomics can identify key disease-related genes and reveal changes in metabolic pathways and networks in organisms, thereby enabling a comprehensive understanding of molecular changes during the occurrence and development of diseases. To explore the molecular changes associated with inflammatory aging, we performed transcriptomic and metabolomic analyses on brain tissues and serum. Transcriptomic analysis showed that the expression levels of *Pstpip2, C1qtnf4, Omg, Zfp36l2, Ankyrin, Pls3, Sytl2, Mex3b* and *Vmp* genes in the brain tissues of aged mice were significantly decreased after CPP treatment. This may activate the MAPK signaling pathway directly or indirectly to regulate the occurrence and development of chronic inflammation. In the KEGG pathway analysis of the serum metabolome, the MAPK signaling pathway was significantly enriched. Through the integrated analysis of metabolomics and transcriptomics data, it was inferred that the MAPK signaling pathway may be a crucial mediator of the anti-aging effects of CPP. The mitogen-activated protein kinase (MAPK) family comprises critical intracellular signaling molecules that orchestrate diverse cellular cascades. Key members include ERK1/2, JNK1/2/3, p38-MAPK isoforms, and the ERK signaling axis, each of which governs distinct physiological and pathological processes through phosphorylation-dependent mechanisms [37]. These pathways play fundamental regulatory roles in the process of cell aging, influencing various cellular functions, such as mitochondrial function, fatty acid and cholesterol synthesis, and inflammation regulation [38,39]. Multiple studies have emphasized the importance of the MAPK pathway in the aging process. For example, mTORC1 activation can augment the protein synthesis of MKK6 and enhance the activation of the p38 MAPK-p53 pathway, thereby counteracting the decline in the structure and function of intestinal villi associated with aging [40]. Additionally, certain plant extracts, such as the methanol extracts of *Malus baccata* [41], resveratrol [42] and *Artocarpus altilis* [43], can reduce ROS production via the MAPK pathway, thereby slowing skin aging. The activity of p38α-MAPK in myeloid cells can impact the immune response and the clearance of Aβ, which is closely associated with cognitive function and neurodegenerative diseases during the aging process [44]. The JNK pathway can regulate liver homeostasis during aging [45] and mediate glycolysis-induced spermatogonial stem cell aging [46]. The administration of erythropoietin in aging rats enhances the ERK/Nrf2-Are pathway, reducing oxidative stress levels and conferring protective effects against aging [47]. Importantly, these studies emphasize the crucial role of the MAPK pathway in the aging process. Our Western blotting (WB) analysis revealed that CPP notably reduces the phosphorylation of JNK and P38 but notably increases that of ERK. Thus, CPP clearly has anti-inflammatory and anti-aging effects through MAPK pathway modulation.

The gut microbiota, aging, and inflammation interact with each other, forming a vicious cycle. The aging process initiates gut microbial imbalance, which subsequently drives inflammatory responses. These inflammatory cascades reciprocally amplify age-related physiological deterioration, forming a self-reinforcing cycle between microbiota disruption and senescence. Inflammation damages the intestinal mucosa and microbiota through oxidative stress, exacerbating dysbiosis, impairing various organ systems, and accelerating the decline of physical functions [48,49,50,51]. Therefore, we conducted a study to examine the implications of CPP for both cognition and gut microbiota. 16S rRNA sequencing revealed that CPP alleviated gut microbiota disorders in aged mice. By leveraging metagenomic profiling of the intestinal microbiota, CPP ameliorates microbial imbalance through enrichment of SCFA-producing bacteria and enhancement of community stability. Pearson correlation analysis indicated that the gut microbiota was related to inflammation and oxidative stress. Research indicates that *Akkermansia muciniphila* enhances short-chain fatty acid (SCFA) production, stimulates regulatory T-cell differentiation, and modulates immune responses while exhibiting an inverse association with inflammatory markers [52,53]. It is important to note that the interaction between gut microbiota and inflammation is bidirectional: gut microbiota dysbiosis can trigger or exacerbate inflammatory responses, while chronic inflammation can, in turn, disrupt gut microbiota homeostasis, creating a vicious cycle that contributes to aging-related decline. Its abundance is low in patients with inflammatory diseases. Supplementation with *Akkermansia muciniphila* increases the acetic acid content in the intestines of elderly mice [28,54,55]. Our findings demonstrated that CPP markedly elevated *Akkermansia muciniphila* levels and acetic acid concentrations, thereby modulating intestinal inflammatory pathways and mitigating age-related physiological decline. Additionally, the gut microbiota impacts the host’s neural function and behavior through the gut–brain pathway. Its metabolites influence neurotransmitters and the neuroendocrine system, affecting cognition and emotions [56,57]. Therefore, CPP-mediated regulation of the gut microbiota may be an important way to improve the cognition of aging mice, though this potential mechanism also requires further validation to clarify its link with inflammatory modulation.

Our study revealed a vicious cycle among the gut microbiota, aging, and inflammation. Aging initiates gut microbiota dysbiosis, which exacerbates inflammation, and inflammation, in turn, accelerates the aging process. Given this, it is imperative to intervene in this tripartite relationship. Our results demonstrate that CPP can regulate the gut microbiota, strengthen intestinal barrier function, and inhibit the growth of harmful bacteria, thereby alleviating inflammation. This also contributes to maintaining the stability of the gut microbiota, reducing the inflammatory response, and ultimately delaying the aging process. Overall, the profound connection between the gut microbiota, aging, and inflammation underscores the potential for new strategies to promote health and slow the effects of aging.

Despite the valuable contributions of this research, certain limitations persist. First, this research was conducted solely in animal models. Although animal models can partially mimic the human aging process, there are still inherent differences from the human physiological state. Future research should prioritize the performance of clinical trials to validate the efficacy and safety of CPP in human aging-related diseases, thereby providing a more robust foundation for its clinical application. Second, while transcriptomics, metabolomics, and gut microbiota analyses have provided valuable insights into the anti-aging mechanism of CPP, the precise molecular regulatory network remains to be fully elucidated. For example, the specific targets of CPP within the MAPK signaling pathway have not been definitively identified, and the interaction mechanism between CPP and gut microbiota metabolites requires further in-depth investigation. Future research could employ advanced molecular biology techniques, such as gene knockout, overexpression, and CRISPR-Cas9 gene editing, combined with sophisticated bioinformatics analysis, to explore the comprehensive molecular mechanism of CPP. This would enable the clarification of its intracellular signal transduction pathways and regulatory networks.

In conclusion, this research comprehensively examined the protective effects of CPP on aging mice, exploring various factors such as cognitive function, systemic aging markers, transcriptomics, metabolomics, and gut microbiota. The results indicate that CPP may enhance multiple aspects of aging and could exert its anti-aging effects via the MAPK pathway. Future studies should aim to pinpoint the specific targets of CPP within this pathway and conduct thorough clinical trials to assess its potential for treating age-related diseases in humans. Additionally, more investigation is needed to understand how CPP affects gut microbiota and the aging process at a molecular level. These findings lay a solid theoretical groundwork for developing anti-aging medications or health products based on CPP.

## 4. Materials and Methods

### 4.1. Animals

Male C57BL/6 mice (6-week-old specific pathogen-free [SPF] grade, 18–22 g body weight) were obtained from Beijing Vital River Laboratory Animal Technology Co., Ltd. (License SCXK [Jing] 2021-0006, Beijing, China). The experimental protocols adhered to the Guide for the Care and Use of Laboratory Animals and received ethical approval (No. SLXD-20230425019, approved on 12 December 2025) from the Animal Ethics Committee of the Institute of Medicinal Plant Development, Chinese Academy of Medical Sciences.

### 4.2. Drugs and Reagents

D-Galactose (product number WXBD9150V) was purchased from SIGMA-ALDRICH (Germany); *Codonopsis pilosula* polysaccharides (CPPs) were obtained from GLPBIO (batch number GB10780, purity: >85.00%); Donepezil (batch number GC30810) was purchased from GLPBIO; kits for TNF-α, IL-1β, IL-18, SOD, MDA, GSH-Px, and CAT (product numbers HY-H0019, HY-H0001, HY-H0014, HY-M0001, HY-M0003, HY-M0004, and HY-M0018) were purchased from Beijing Huaying Biotechnology Research Institute (Beijing, China). Hematoxylin and eosin (HE) staining kits and Nissl staining kits (product numbers G1120 and G1430) were purchased from Solarbio (San Diego, CA, USA). Primary antibodies against BDNF, NF-κB, and TNF-α (product numbers K008206P, K112437P, and K108751P) were purchased from Solarbio.

### 4.3. Instruments

The following instruments were used: BSA224S-CW Electronic Balance (Sartorius, Germany); Water Maze and Water Maze Behavioral Video Analysis System (Shanghai Xinruan Information Technology Co., Ltd., Shanghai, China); DR-200BS Microplate Reader (Wuxi Huawei Delang Instrument Co., Ltd., Wuxi, Jiangsu, China); P250 FLASH Panoramic Scanner (3DHISTECH, Budapest, Hungary); X-Cite120 PC Q Fluorescence Microscope (Excelitas, Malvern, PA, USA); and 5424R Centrifuge (Eppendorf, Enfield, CT, USA).

### 4.4. Animal Grouping and Drug Administration

Thirty mice were randomly assigned to five experimental cohorts *(n* = 6 per group). The groups were the Cont group, the model group (200 mg·kg^−1^ D-gal), the low-dose CPP group (200 mg·kg^−1^, CPP-L group), the high-dose CPP group (400 mg·kg^−1^, CPP-H group), and the donepezil group (3 mg·kg^−1^ donepezil, DON group). All the groups underwent a 1-week acclimatization period with daily intragastric gavage. The model, CPP-L, CPP-H, and DON groups subsequently received subcutaneous D-gal injections (200 mg/kg/day) for 8 weeks, alongside continued oral administration. The control animals received equivalent volumes of saline via the same route.

### 4.5. Morris Water Maze Experiment

The Morris water maze test was conducted 48 h posttreatment. The apparatus consisted of a circular pool divided into four equal quadrants maintained at 25 ± 2 °C. To obscure the submerged platform, titanium dioxide was dispersed uniformly in the water. The protocol comprised two phases: learning and testing. During the learning phase, the mice were introduced into random quadrants and allowed 60 s to locate the submerged platform. If unsuccessful, they were guided to the platform and permitted to remain for 10 s. This training regimen was repeated daily over four consecutive days. During the experimental assessment, the platform was withdrawn, and the spatial navigation capabilities of the mice were analyzed using VisuTrack software.(V3.0.0.1) Parameters such as the frequency of crossings at the prior platform location, duration spent in the designated quadrant, and escape delay within a 60-s interval were quantified. These metrics collectively provide insights into the spatial learning proficiency and memory retention of rodents.

### 4.6. Enzyme-Linked Immunosorbent Assay (ELISA)

Following euthanasia via ocular enucleation in accordance with AVMA guidelines, whole blood samples were immediately collected and processed through centrifugation at 3000× *g* (corresponding to 3000 rpm using a rotor radius of 10 cm) for 10 min at 4 °C. The resulting serum supernatant was aliquoted and stored at−80 °C until subsequent biochemical analyses. The levels of oxidative stress markers (SOD, MDA, GSH-Px, and CAT) and proinflammatory cytokines (TNF-α, IL-1β, and IL-18) were quantified using ELISA kits (Beijing SINO-UK Institute of Biological Technology, Beijing, China). After drug administration, the cells were collected and rinsed three times with PBS. Then, the mixture was centrifuged at 1000 r/min for 5 min to obtain the cell pellet. The remaining methods were the same as those used for the serum ELISA.

### 4.7. Histopathological Assessment by HE Staining

To evaluate histopathological changes, three mice per group were randomly selected and euthanized after blood collection. Multiple organ specimens (cerebral, pulmonary, hepatic, intestinal, and muscular tissues) were subjected to standardized histopathological processing: primary immobilization in 10% phosphate-buffered formalin (pH 7.4), a graded ethanol dehydration series (70–100% *v*/*v*), xylene-mediated transparency enhancement, and final encapsulation within paraffin blocks. Tissue sections 2-4 μm thick were prepared, deparaffinized, and stained with HE. After mounting, histopathological alterations in the hippocampus, cerebral cortex, and other tissues were observed under a light microscope and photographed.

### 4.8. Immunofluorescence to Detect Changes in the Expression of BDNF, GFAP1, and IBA1 in Brain Tissue

The sections were prepared using the same method as for HE staining, while the subsequent steps followed the protocol for immunohistochemistry. The BDNF, GFAP1, and IBA1 antibodies were incubated with the samples at 4 °C overnight. After the primary antibodies were removed, the cells were incubated with the secondary antibodies labeled with luciferase in the dark for 1 h. After the samples were washed with PBS, the nuclei were labeled with DAPI for 5 min. Subsequently, an anti-fade mounting medium was applied, and fluorescent signals were analyzed and captured under a microscope in the dark.

### 4.9. Transcriptomics

Transcriptome sequencing was conducted in accordance with the methodology outlined by Lin [58]. For the transcriptomic analysis, three biological replicates of brain tissue samples were prepared for each experimental group. The data generated from Illumina NovaSeq 6000 sequencing were subsequently transformed into sequence data. Following data filtration, the sequences were aligned to the ribosomal database corresponding to the species under investigation. HISAT2 software (V2.2.1) facilitated the alignment analysis. Subsequently, StringTie (v2.2.1) was utilized for transcript reconstruction, whereas RSEM (v1.3.3) was employed to quantify the expression levels of all genes within each sample. To assess the reproducibility of expression levels across samples, principal component analysis (PCA) and calculation of the Pearson correlation coefficient were performed, leading to the exclusion of any outlier samples. Differentially expressed genes (DEGs) were screened using a threshold of |log2-fold change| > 1.5 and *p* < 0.05. These DEGs were subsequently subjected to functional enrichment analyses, including Gene Ontology (GO) annotation and Kyoto Encyclopedia of Genes and Genomes (KEGG) pathway mapping, to explore their biological roles.

### 4.10. Metabolomics

Serum samples were thawed at 4 °C and extracted using a pre-cooled methanol:acetonitrile:water (2:2:1, *v*/*v*/*v*). After vortex-mixing, the mixtures were ultrasonicated at low temperature (40 kHz, 30 min), incubated at −20 °C for 10 min, and centrifuged (14,000× *g*, 20 min, 4 °C). The supernatant was lyophilized and reconstituted in acetonitrile/water (1:1, *v*/*v*) and centrifuged again before LC-MS analysis. Chromatographic separation was performed by an Agilent 1290 Infinity II UHPLC system with a ZORBAX HILIC Plus column (2.1 × 100 mm, 1.8 μm (Agilent Technologies, Santa Clara, CA, USA)). High-resolution mass spectrometric data were acquired on an AB SCIEX TripleTOF 6600 platform (AB Sciex, Framingham, MA, USA) in dual-polarity ESI mode. The raw files were converted to .mzML format and processed with XCMS for peak detection and alignment. Missing values (>50% within a group) were removed; remaining missing data were imputed (k-NN, k = 5). Data were normalized to total peak area and analyzed by OPLS-DA. Metabolites were annotated against MassBank, Metlin, MoNA, and an in-house MS/MS library. Final data were z-score normalized and clustered using hierarchical clustering.

### 4.11. 16S Ribosomal RNA Gene Sequencing Analysis

The 16S rRNA gene, present in prokaryotes but absent in mammalian genomes, was used to assess the gut microbiota taxonomic structure in intestinal samples. Genomic DNA was isolated using HiPure kits (Magen Biotechnology, Guangzhou, China). The primers Arch519F and Arch915R with 8-base barcodes were used. The V3–V4 region of 16S rDNA was amplified using PCR. The PCR amplicons were separated on a 2% agarose gel, purified with an AxyPrep kit (Axygen, Corning Life Sciences, Union City, CA, USA), and quantified with ABI StepOnePlus(Applied Biosystems, Foster City, CA, USA). Equimolar amplicons were pooled and sequenced (2 × 250) on an Illumina platform following the manufacturer’s protocols. Guangzhou Gene Denovo Biotechnology Co., Ltd. (Guangzhou, China) assisted with 16S rRNA sequencing.

After obtaining raw reads through sequencing, we first filtered low-quality reads using the FASTP software (V0.23.2). Paired-end reads were combined to extend the read length using FLASH software (V1.2.11) and spliced into tag sequences, which were then filtered to obtain clean tags. Next, based on the clean tags, the UPARSE algorithm of USEARCH software (V11.0.667) was adopted to create and cluster OTUs from the sequences when the sequence similarity was higher than 0.97. After obtaining the OTUs, OTU abundance statistics were performed based on the effective tags. Additionally, species composition, α-diversity, and β-diversity analyses were conducted to indicate species richness and inter-group species diversity. The pheatmap package (version 1.0.12) in the R language was used for clustering and heatmap analysis of gut microbiota at the phylum and genus levels.

### 4.12. RT-qPCR

Total RNA extraction from cerebral tissues and cellular specimens was performed using TransZol. cDNA synthesis was subsequently conducted with PrimeScript™ RT Master Mix (Takara Bio, San Jose, CA, USA) under optimized thermal cycling conditions (37 °C for 15 min → 85 °C for 5 s). For quantitative gene expression analysis, TB Green^®^ Premix Ex Taq™ II (Takara Bio) was used on a QuantStudio 6 Pro system, with three technical replicates per sample. All primer pairs (sequences detailed in Table 1) demonstrated >95% amplification efficiency through standard curve validation and were designed using NCBI Primer-BLAST with melting temperatures between 58 °C and 62 °C. RNA integrity was verified using a Bioanalyzer 2100(Agilent Technologies Inc., Santa Clara, United States) (RIN > 8.0) prior to reverse transcription. All procedures adhered to the manufacturer’s guidelines.

### 4.13. Western Blot Analysis

An appropriate amount of brain tissue and cells was added to RIPA lysis buffer containing PMSF and phosphatase inhibitors, and the tissue was homogenized. The mixture was lysed at 4 °C for 1 h and then centrifuged at 12,000 r/min for 15 min (centrifugal radius of 8.4 cm), after which the total protein in the supernatant was collected. The protein concentration was determined using the BCA method. A total of 20 μg of total protein in each group was removed, the proteins were separated using 10% SDS-PAGE, and the proteins were transferred to an NC membrane using electrotransfer. The samples were blocked with 5% skim milk at RT for 2 h, after which primary antibodies against p-ERK (1:1000), ERK (1:1000), p-JNK (1:1000), JNK (1:1000), p-P38 MAPK (1:500), P38 MAPK (1:1000), and FOXO1 (1:1000) were added, and the samples were incubated overnight at 4 °C. The samples were washed with TBST 3 times for 10 min each. Horseradish peroxidase-labeled secondary antibody was added, the membrane was incubated at RT for 2 h, and the membrane was washed with TBST 3 times for 10 min each. The images were developed using the enhanced chemiluminescence (ECL) method, and ImageJ software(V1.53) was used to calculate the gray value of each band. β-Actin (1:5000) was used as the internal reference, and the relative expression level of the target protein was calculated.

### 4.14. Statistical Processing Methods

Statistical analyses were performed using SPSS 22.0. The quantitative data are expressed as the means ± standard deviations (means ± SEM). Differences among multiple groups were assessed by one-way ANOVA, whereas pairwise comparisons were conducted using unpaired Student’s t-tests and least significant difference (LSD) tests. A *p*-value < 0.05 was considered statistically significant.

## Figures and Tables

**Table 1:** Primer sequences used in the experiment.

Primer	5′ to 3′
18 s-F	GTAACCCGTTGAACCCCATT
18 s-R	CCATCCAATCGGTAGTAGCG
P53-F	GTCACAGCACATGACGGAGG
P53-R	TCTTCCAGATGCTCGGGATAC
P21-F	CCTGGTGATGTCCGACCTG
P21-R	CCATGAGCGCATCGCAATC
P16-F	CGCAGGTTCTTGGTCACTGT
P16-R	TGTTCACGAAAGCCAGAGCG

## Data Availability

The original contributions presented in this study are included in the article. Further inquiries can be directed to the corresponding author.

## Supplementary Materials

The following supporting information can be downloaded at: https://www.mdpi.com/article/10.3390/ijms27093933/s1.
