# Supplementary material for: Codonopsis pilosula Polysaccharide (CPP) Alleviates D-Gal-Induced Aging and Gut Microbiota Dysbiosis
Source: Int J Mol Sci. 2026 Apr 28;27(9):3933. doi: 10.3390/ijms27093933 (PMC13164561; doi:10.3390/ijms27093933)
Supplement: Supplementary file 1 [file ijms-27-03933-s001.zip › ijms-4095164-supplementary.pdf]

Codonopsis pilosula Polysaccharide (CPP) Alleviates D-Gal-Induced Aging and Gut Microbiota Dysbiosis

Bin Zhang [1,2,3,4,†], Chongyang Zhang [1,2,3,4,5,†], Miao Yu [1,2,3,4], Yudie Zhang [1,2,3,4], Xiangming Wang [5], Rongchang Chen [1,*] and Xiaobo Sun [1,2,3,4,*]

Supplementary Figures

Supplementary Figure S1. CPP can significantly improve intestinal permeability and inflammation in D - gal - induced senescent mice.

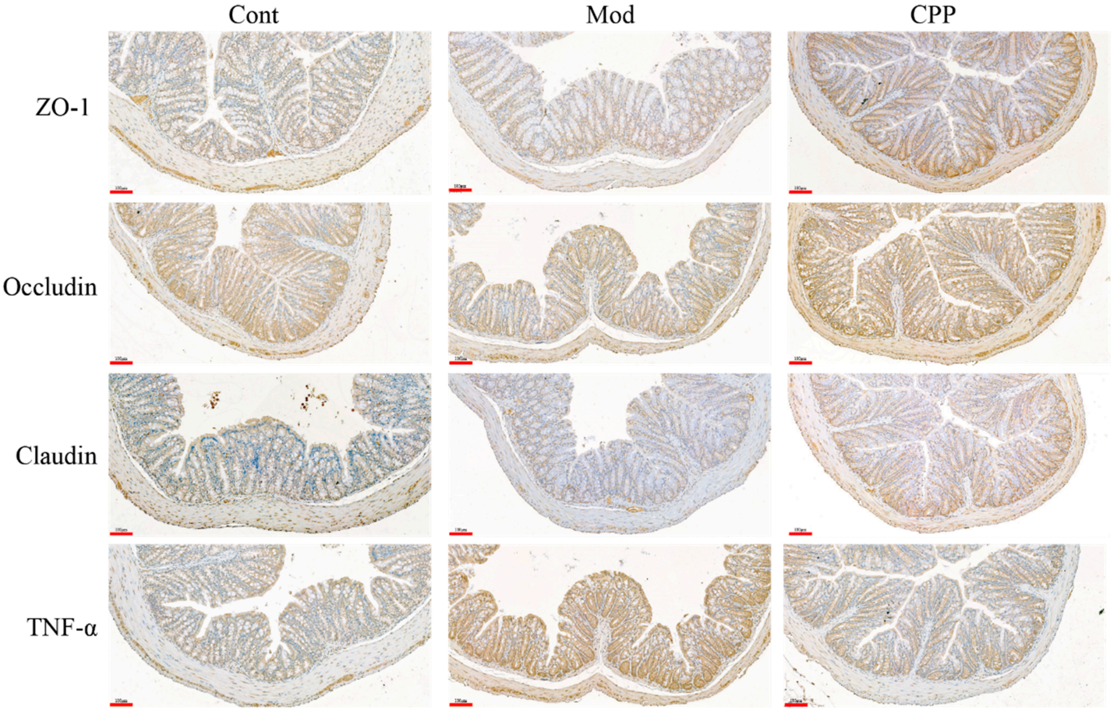

Fig.S1 Effects of CPP on tight junction proteins and inflammatory levels in the intestinal tissues of senescent mice. Immunohistochemical staining of the intestinal tissues shows the distribution of ZO-1, Occludin, Claudin, and TNF-*α* in the intestinal tissues of the Control (Cont), Model (Mod), and High-dose CPP (CPPH) groups.（n=3）

Supplementary Figure S2. CPP can significantly improve intestinal permeability and inflammation in D - gal - induced

senescent mice.

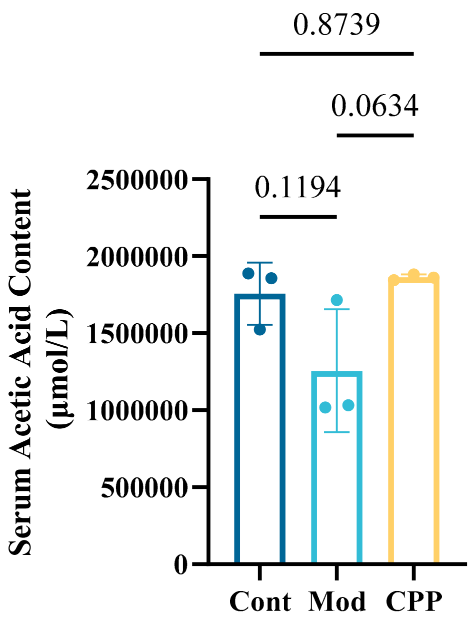

Fig.S2 Effects of CPP on the level of acetic acid in the serum. The expression level of acetic acid in the non-targeted metabolomics of the serum. (n = 3)

Supplementary Figure S3. HPLC Analysis of Monosaccharides from the Hydrolysis of Codonopsis Polysaccharides.

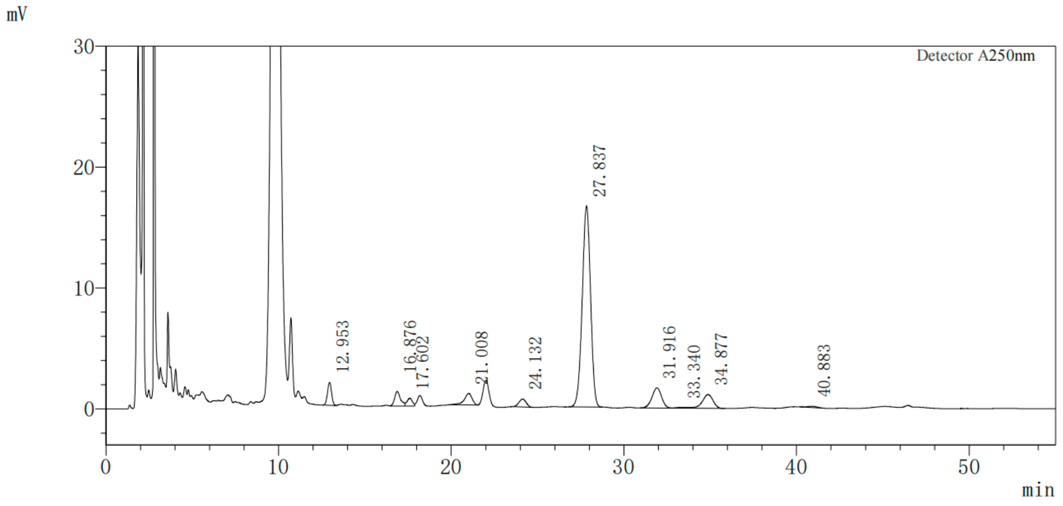

Fig.S3 HPLC Analysis of Monosaccharides from the Hydrolysis of Codonopsis Polysaccharides.. The analysis of monosaccharide components from the hydrolysis of Codonopsis polysaccharides was performed using HPLC. The x-axis represents the retention time (min), and the y-axis represents the signal intensity (mV). The main peaks correspond to the retention times of 12.953 min (Mannose), 21.008 min (Glucuronic acid), 24.132 min (Galacturonic acid), 27.837 min (Glucose), 31.916 min (Galactose), 33.340 min

(Xylose), 34.877 min (Arabinose), and 40.883 min (Fucose).

Supplementary Table S1. Analysis of Retention Time, Peak Area, Peak Height, and Area Percentage of Monosaccharides from the Hydrolysis of Codonopsis Polysaccharides

Number	Attention Time	Compound Name	Area	Height	Area%
1	12.953	Mannose	30918	1901	3.843
2	16.876	Ribose	28762	1211	3.575
3	17.602	Rhamnose	14279	657	1.775
4	21.008	Glucuronic acid	30461	955	3.786
5	24.132	Galacturonic acid	19211	657	2.388
6	27.837	Glucose	558011	16609	69.362
7	31.916	Galactose	65331	1669	8.121
8	33.340	Xylose	4028	74	0.501
9	34.877	Arabinose	49480	1151	6.150
10	40.883	Fucose	4013	108	0.499
**Sum**			**804493**	**24992**	**100.000**

Supplementary Table S2. KEGG Enrichment and Correlation Analysis of Serum Metabolome and Brain Transcriptome in CPP Group

KEGG_A_class	KEGG_B_class	Pathway	Gene_All (9181)	Metabolite_All (959)
Metabolism	Global and overview maps	Metabolic pathways	1635	611
Environmental Information Processing	Membrane transport	ABC transporters	54	68
Metabolism	Global and overview maps	Biosynthesis of amino acids	80	58
Organismal Systems	Digestive system	Bile secretion	104	57
Metabolism	Lipid metabolism	Steroid hormone biosynthesis	100	42
Environmental Information Processing	Signaling molecules and interaction	Neuroactive ligand-receptor interaction	389	39
Metabolism	Nucleotide metabolism	Purine metabolism	137	36
Metabolism	Nucleotide metabolism	Pyrimidine metabolism	57	34
Metabolism	Amino acid metabolism	Tryptophan metabolism	52	32
Metabolism	Global and overview maps	2-Oxocarboxylic acid metabolism	20	32
Metabolism	Lipid metabolism	Arachidonic acid metabolism	89	31
Metabolism	Global and overview maps	Carbon metabolism	121	31
Metabolism	Amino acid metabolism	Cysteine and methionine metabolism	55	29

Organismal Systems	Digestive system	Vitamin digestion and absorption	25	28
Metabolism	Carbohydrate metabolism	Amino sugar and nucleotide sugar metabolism	51	27
Metabolism	Amino acid metabolism	Glycine, serine and threonine metabolism	42	25
Metabolism	Xenobiotics biodegradation and metabolism	Drug metabolism - cytochrome P450	73	24
Human Diseases	Cancer: overview	Central carbon metabolism in cancer	71	23
Metabolism	Amino acid metabolism	Arginine and proline metabolism	55	22
Metabolism	Amino acid metabolism	Histidine metabolism	27	22
Metabolism	Lipid metabolism	Glycerophospholipid metabolism	99	22
Metabolism	Amino acid metabolism	Tyrosine metabolism	39	21
Metabolism	Lipid metabolism	Biosynthesis of unsaturated fatty acids	35	21
Organismal Systems	Digestive system	Protein digestion and absorption	109	21
Metabolism	Carbohydrate metabolism	Galactose metabolism	34	20
Organismal Systems	Nervous system	Serotonergic synapse	138	20
Metabolism	Carbohydrate metabolism	Pentose phosphate pathway	33	18
Metabolism	Amino acid metabolism	Lysine degradation	65	18
Metabolism	Metabolism of other amino acids	beta-Alanine metabolism	33	18
Genetic Information Processing	Translation	Aminoacyl-tRNA biosynthesis	44	18
Metabolism	Carbohydrate metabolism	Ascorbate and aldarate metabolism	33	17
Metabolism	Lipid metabolism	Primary bile acid biosynthesis	18	17

Environmental Information Processing	Signal transduction	cAMP signaling pathway	221	17
Human Diseases	Cancer: overview	Pathways in cancer	549	17
Human Diseases	Cancer: overview	Choline metabolism in cancer	101	17
Metabolism	Amino acid metabolism	Alanine, aspartate and glutamate metabolism	38	16
Organismal Systems	Sensory system	Taste transduction	91	16
Metabolism	Metabolism of cofactors and vitamins	Nicotinate and nicotinamide metabolism	42	15
Metabolism	Metabolism of cofactors and vitamins	Ubiquinone and other terpenoid-quinone biosynthesis	12	14
Metabolism	Amino acid metabolism	Phenylalanine, tyrosine and tryptophan biosynthesis	8	14
Metabolism	Metabolism of other amino acids	Glutathione metabolism	71	14
Metabolism	Metabolism of cofactors and vitamins	Pantothenate and CoA biosynthesis	22	14
Metabolism	Metabolism of cofactors and vitamins	Porphyrin metabolism	44	14
Organismal Systems	Nervous system	Retrograde endocannabinoid signaling	149	14
Organismal Systems	Sensory system	Inflammatory mediator regulation of TRP channels	132	14
Metabolism	Biosynthesis of other secondary metabolites	Caffeine metabolism	6	13

Organismal Systems	Endocrine system	Ovarian steroidogenesis	69	13
Metabolism	Amino acid metabolism	Arginine biosynthesis	20	12
Metabolism	Amino acid metabolism	Phenylalanine metabolism	20	12
Metabolism	Carbohydrate metabolism	Glyoxylate and dicarboxylate metabolism	31	12
Metabolism	Metabolism of cofactors and vitamins	Folate biosynthesis	29	12
Organismal Systems	Endocrine system	Aldosterone synthesis and secretion	105	12
Human Diseases	Endocrine and metabolic disease	Insulin resistance	112	12
Organismal Systems	Digestive system	Mineral absorption	54	12
Metabolism	Metabolism of other amino acids	Taurine and hypotaurine metabolism	22	11
Metabolism	Lipid metabolism	alpha-Linolenic acid metabolism	25	11
Environmental Information Processing	Signal transduction	Sphingolipid signaling pathway	125	11
Organismal Systems	Endocrine system	Glucagon signaling pathway	105	11
Metabolism	Carbohydrate metabolism	Glycolysis / Gluconeogenesis	69	10
Metabolism	Carbohydrate metabolism	Fructose and mannose metabolism	37	10
Metabolism	Lipid metabolism	Fatty acid biosynthesis	19	10
Metabolism	Lipid metabolism	Steroid biosynthesis	20	10

Metabolism	Biosynthesis of other secondary metabolites	Monobactam biosynthesis	2	10
Metabolism	Amino acid metabolism	Valine, leucine and isoleucine biosynthesis	4	10
Metabolism	Biosynthesis of other secondary metabolites	Neomycin, kanamycin and gentamicin biosynthesis	5	10
Metabolism	Lipid metabolism	Linoleic acid metabolism	55	10
Metabolism	Lipid metabolism	Sphingolipid metabolism	49	10
Organismal Systems	Endocrine system	Thyroid hormone synthesis	73	10
Metabolism	Carbohydrate metabolism	Pentose and glucuronate interconversions	37	9
Metabolism	Amino acid metabolism	Valine, leucine and isoleucine degradation	57	9
Metabolism	Amino acid metabolism	Lysine biosynthesis	1	9
Metabolism	Carbohydrate metabolism	Starch and sucrose metabolism	36	9
Metabolism	Carbohydrate metabolism	Butanoate metabolism	28	9
Metabolism	Metabolism of cofactors and vitamins	Thiamine metabolism	16	9
Environmental Information Processing	Signal transduction	AMPK signaling pathway	126	9
Organismal Systems	Circulatory system	Vascular smooth muscle contraction	144	9
Organismal Systems	Immune system	Fc epsilon RI signaling pathway	191	9
Organismal Systems	Endocrine system	Prolactin signaling pathway	83	9

Organismal Systems	Endocrine system	Regulation of lipolysis in adipocytes	60	9
Organismal Systems	Digestive system	Carbohydrate digestion and absorption	50	9
Human Diseases	Neurodegenerative disease	Parkinson disease	274	9
Metabolism	Carbohydrate metabolism	C5-Branched dibasic acid metabolism	1	8
Metabolism	Metabolism of cofactors and vitamins	Vitamin B6 metabolism	9	8
Metabolism	Metabolism of terpenoids and polyketides	Terpenoid backbone biosynthesis	24	8
Environmental Information Processing	Signal transduction	Phospholipase D signaling pathway	273	8
Cellular Processes	Cellular community - eukaryotes	Gap junction	88	8
Organismal Systems	Immune system	Platelet activation	128	8
Organismal Systems	Nervous system	Synaptic vesicle cycle	77	8
Human Diseases	Cancer: specific types	Prostate cancer	101	8
Metabolism	Carbohydrate metabolism	Inositol phosphate metabolism	73	7
Metabolism	Carbohydrate metabolism	Pyruvate metabolism	45	7
Metabolism	Metabolism of cofactors and vitamins	Biotin metabolism	3	7
Metabolism	Energy metabolism	Sulfur metabolism	11	7

Environmental Information Processing	Signal transduction	HIF-1 signaling pathway	117	7
Environmental Information Processing	Signal transduction	Phosphatidylinositol signaling system	98	7
Organismal Systems	Immune system	Fc gamma R-mediated phagocytosis	216	7
Organismal Systems	Endocrine system	Insulin secretion	87	7
Organismal Systems	Endocrine system	Oxytocin signaling pathway	155	7
Organismal Systems	Endocrine system	Renin secretion	76	7
Human Diseases	Infectious disease: parasitic	African trypanosomiasis	158	7
Human Diseases	Infectious disease: parasitic	Amoebiasis	232	7
Metabolism	Carbohydrate metabolism	Citrate cycle (TCA cycle)	32	6
Metabolism	Energy metabolism	Oxidative phosphorylation	138	6
Metabolism	Metabolism of cofactors and vitamins	One carbon pool by folate	19	6
Metabolism	Metabolism of cofactors and vitamins	Riboflavin metabolism	8	6
Metabolism	Xenobiotics biodegradation and metabolism	Drug metabolism - other enzymes	99	6
Organismal Systems	Circulatory system	Adrenergic signaling in cardiomyocytes	148	6

Organismal Systems	Nervous system	Cholinergic synapse	114	6
Organismal Systems	Nervous system	Dopaminergic synapse	134	6
Organismal Systems	Nervous system	Long-term depression	62	6
Organismal Systems	Sensory system	Phototransduction - fly	30	6
Organismal Systems	Endocrine system	GnRH signaling pathway	94	6
Human Diseases	Endocrine and metabolic disease	AGE-RAGE signaling pathway in diabetic complications	103	6
Organismal Systems	Digestive system	Salivary secretion	88	6
Metabolism	Lipid metabolism	Glycerolipid metabolism	65	5
Metabolism	Carbohydrate metabolism	Propanoate metabolism	31	5
Metabolism	Metabolism of cofactors and vitamins	Retinol metabolism	104	5
Metabolism	Xenobiotics biodegradation and metabolism	Metabolism of xenobiotics by cytochrome P450	81	5
Organismal Systems	Environmental adaptation	Circadian entrainment	101	5
Organismal Systems	Nervous system	Neurotrophin signaling pathway	125	5
Organismal Systems	Endocrine system	Estrogen signaling pathway	137	5

Organismal Systems	Digestive system	Gastric acid secretion	76	5
Organismal Systems	Digestive system	Fat digestion and absorption	44	5
Human Diseases	Substance dependence	Alcoholism	202	5
Human Diseases	Infectious disease: parasitic	Leishmaniasis	191	5
Metabolism	Lipid metabolism	Fatty acid degradation	53	4
Metabolism	Metabolism of terpenoids and polyketides	Insect hormone biosynthesis	4	4
Metabolism	Global and overview maps	Fatty acid metabolism	62	4
Environmental Information Processing	Signal transduction	Ras signaling pathway	239	4
Environmental Information Processing	Signal transduction	Calcium signaling pathway	366	4
Genetic Information Processing	Folding, sorting and degradation	Sulfur relay system	8	4
Environmental Information Processing	Signal transduction	VEGF signaling pathway	60	4
Organismal Systems	Nervous system	Long-term potentiation	67	4
Organismal Systems	Nervous system	Glutamatergic synapse	116	4

Organismal Systems	Endocrine system	Melanogenesis	101	4
Organismal Systems	Endocrine system	Thyroid hormone signaling pathway	121	4
Organismal Systems	Endocrine system	Adipocytokine signaling pathway	71	4
Organismal Systems	Excretory system	Endocrine and other factor-regulated calcium reabsorption	62	4
Organismal Systems	Digestive system	Pancreatic secretion	114	4
Human Diseases	Substance dependence	Cocaine addiction	49	4
Human Diseases	Substance dependence	Amphetamine addiction	69	4
Human Diseases	Cancer: overview	Chemical carcinogenesis - DNA adducts	93	4
Human Diseases	Cancer: specific types	Non-small cell lung cancer	72	4
Human Diseases	Immune disease	Asthma	148	4
Environmental Information Processing	Signal transduction	MAPK signaling pathway	301	3
Environmental Information Processing	Signal transduction	ErbB signaling pathway	87	3
Environmental Information Processing	Signal transduction	Rap1 signaling pathway	222	3

Environmental Information Processing	Signal transduction	cGMP-PKG signaling pathway	173	3
Organismal Systems	Immune system	Chemokine signaling pathway	193	3
Environmental Information Processing	Signal transduction	NF-kappa B signaling pathway	230	3
Environmental Information Processing	Signal transduction	FoxO signaling pathway	134	3
Cellular Processes	Transport and catabolism	Autophagy - animal	147	3
Cellular Processes	Transport and catabolism	Lysosome	142	3
Organismal Systems	Aging	Longevity regulating pathway - worm	90	3
Organismal Systems	Immune system	Natural killer cell mediated cytotoxicity	240	3
Organismal Systems	Immune system	T cell receptor signaling pathway	109	3
Organismal Systems	Immune system	B cell receptor signaling pathway	200	3
Organismal Systems	Nervous system	GABAergic synapse	90	3
Organismal Systems	Excretory system	Aldosterone-regulated sodium reabsorption	39	3

Organismal Systems	Excretory system	Proximal tubule bicarbonate reclamation	22	3
Human Diseases	Neurodegenerative disease	Amyotrophic lateral sclerosis	382	3
Human Diseases	Substance dependence	Morphine addiction	94	3
Human Diseases	Substance dependence	Nicotine addiction	40	3
Human Diseases	Infectious disease: bacterial	Vibrio cholerae infection	51	3
Human Diseases	Infectious disease: bacterial	Shigellosis	255	3
Human Diseases	Cancer: specific types	Glioma	76	3
Metabolism	Metabolism of other amino acids	Selenocompound metabolism	15	2
Metabolism	Glycan biosynthesis and metabolism	Glycosylphosphatidylinositol (GPI)-anchor biosynthesis	26	2
Metabolism	Lipid metabolism	Ether lipid metabolism	49	2
Organismal Systems	Endocrine system	PPAR signaling pathway	92	2
Cellular Processes	Cell growth and death	Oocyte meiosis	122	2
Organismal Systems	Immune system	NOD-like receptor signaling pathway	219	2
Organismal Systems	Sensory system	Phototransduction	27	2
Human Diseases	Endocrine and metabolic disease	Type II diabetes mellitus	49	2
Human Diseases	Neurodegenerative disease	Huntington disease	309	2
Human Diseases	Infectious disease: bacterial	Pathogenic Escherichia coli infection	326	2
Human Diseases	Infectious disease: parasitic	Chagas disease	108	2
Human Diseases	Cancer: specific types	Pancreatic cancer	76	2

Human Diseases	Immune disease	Rheumatoid arthritis	214	2
Metabolism	Lipid metabolism	Fatty acid elongation	30	1
Metabolism	Glycan biosynthesis and metabolism	Glycosaminoglycan biosynthesis - heparan sulfate / heparin	24	1
Metabolism	Metabolism of cofactors and vitamins	Lipoic acid metabolism	3	1
Metabolism	Energy metabolism	Nitrogen metabolism	17	1
Cellular Processes	Transport and catabolism	Endocytosis	267	1
Environmental Information Processing	Signal transduction	mTOR signaling pathway	160	1
Cellular Processes	Cell growth and death	Apoptosis	147	1
Organismal Systems	Aging	Longevity regulating pathway	91	1
Organismal Systems	Development and regeneration	Osteoclast differentiation	128	1
Organismal Systems	Endocrine system	Renin-angiotensin system	38	1
Organismal Systems	Immune system	Intestinal immune network for IgA production	167	1
Organismal Systems	Sensory system	Olfactory transduction	1182	1
Cellular Processes	Cell motility	Regulation of actin cytoskeleton	225	1
Organismal Systems	Endocrine system	Insulin signaling pathway	139	1

Organismal Systems	Endocrine system	Progesterone-mediated oocyte maturation	95	1
Human Diseases	Endocrine and metabolic disease	Non-alcoholic fatty liver disease	159	1
Human Diseases	Neurodegenerative disease	Alzheimer disease	393	1
Human Diseases	Neurodegenerative disease	Prion disease	274	1
Human Diseases	Infectious disease: bacterial	Epithelial cell signaling in Helicobacter pylori infection	70	1
Human Diseases	Infectious disease: bacterial	Pertussis	77	1
Human Diseases	Infectious disease: parasitic	Toxoplasmosis	111	1
Human Diseases	Infectious disease: bacterial	Tuberculosis	304	1
Human Diseases	Infectious disease: viral	Epstein-Barr virus infection	357	1
Human Diseases	Cancer: specific types	Renal cell carcinoma	70	1
Human Diseases	Cancer: specific types	Basal cell carcinoma	63	1
Human Diseases	Cancer: specific types	Small cell lung cancer	96	1
Human Diseases	Immune disease	Autoimmune thyroid disease	197	1
Human Diseases	Immune disease	Systemic lupus erythematosus	266	1
